# Experiences, challenges, and prospects of National Medical Licensing Examination in China

**DOI:** 10.1186/s12909-022-03385-9

**Published:** 2022-05-08

**Authors:** Xiancheng Wang

**Affiliations:** National Medical Examination Center, Beijing, 100097 China

**Keywords:** National Medical Licensing Examination, Medical education, China

## Abstract

Since the implementation of National Medical Licensing Examination (NMLE) system in China, millions of individuals have participated in the examination and have been licensed as physicians. Over the past few decades, NMLE has played a major role in evaluating and guiding Chinese medical education and has made great progress. This commentary discusses the main experience, challenges, and prospects of NMLE in China.

## Background

Universal health is the starting point and foundation of the well-being of all individuals. Without universal health, there cannot be all-round well-being and so all-round modernization cannot be realized. Human beings, especially physicians should develop healthy communities by ensuring sound health for mankind. In the Chinese health service system, healthcare personnel, especially physicians play the most important role. Physicians need to guarantee that they can efficiently play their roles and better deliver high-quality services by ensuring an elaborate training system in medical education, strict qualification authentication and admission, and normative management system. China has gradually established a medical education system with Chinese characteristics, including 5-year, “5 + 3”-year, and 8-year programs. Furthermore, the “5 + 3” model (5-year undergraduate and 3-year residency [standardized residency training]) is the main program in China [1]. The Ministry of Education of the People’s Republic of China has conducted the medical education accreditation for over three decades, and has achieved remarkable success, showing good impact and gaining recognition from the World Federation for Medical Education (WFME) [2].

Medical education is a lifelong learning process with a ladder and gradual stepwise implementation and segmented improvement with different focuses for different stages of life and career of physicians. As the guardians of human health, physicians should be strictly and rigorously prepared for independent practice. The National Medical Licensing Examination (NMLE) is used in many countries to assess whether the applicants for physician qualification have the professional competence necessary for practice [3]. In China, only after passing NMLE can the candidates obtain the physician qualification certificate [4]. The qualification certificate is a label proving that the holder has the relevant competence to independently engage in healthcare services. Furthermore, it is not only an important standard to judge physicians’ professional competence but also an important legal basis for physicians to work at healthcare institutions. The NMLE is being conducted in China since 1998 by the National Medical Examination Center (NMEC), an affiliate unit of the National Health Commission (NHC) of China [5, 6].

NMLE plays an important role in evaluating and guiding Chinese medical education. Periodical reports of NMLE results are submitted to the National Health Commission (NHC) and the Ministry of Education as a reference for decision making. Further, examination results can also be referred back to medical schools and examinees for review so as to help schools improve their teaching and overcome the shortcomings of examinees. The NMLE results are also an important indicator for evaluating medical education quality. If the passing rates of NMLE in one medical school were less than 50% in the recent 3 years, this school will be not eligible to participate in accreditation. Furthermore, the passing rate is also an important reference for getting the accreditation conclusions of medical schools [2, 7]. Moreover, the NMLE syllabus is one of the significant guidelines for conducting education program in medical schools [8]. This commentary summarizes the characteristics and achievements of NMLE in China. It also discusses the main experiences, challenges, and prospects of NMLE.

## Development and achievements of NMLE in China

In June 1998, the People’s Republic of China law on Medical Practitioners was promulgated to legalize the NMLE. Furthermore, NHC was held responsible for the implementation of the NMLE system [4]. The current NMLE consists of two components, the clinical skill examination and the comprehensive medical knowledge examination. The clinical skill examination adopts a 3-station procedure to assess the ability of clinical reasoning, physical examination, and basic clinical operation. The comprehensive medical knowledge examination consists of 600 multiple-choice questions (MCQs), assessing knowledge on basic sciences, clinical medicine, medical humanism, and preventive medicine.

The first ever clinical skill examination of NMLE was held in September 1999. In November of the same year, the first comprehensive medical knowledge examination was held simultaneously at nearly 400 examination centers in 31 examination locations. Since 2001, the clinical skill examination has been administered in July each year, and comprehensive medical knowledge examination has been held nationwide in September each year. The number of candidates appearing for NMLE is large and has increased each year. Since the implementation of this system, more than 13 million individuals have participated in the NMLE and more than 4 million have been licensed as physicians [9]. In 2020, there were a total of 600 examination sites for the comprehensive medical knowledge examination. The passing rates for the clinical skill examination and comprehensive medical knowledge examination among medical students who appeared for NMLE for the first time in 2020 were 88.98 and 72.5%, respectively.

At the same time, the quality of the physicians’ team has undergone tremendous improvement. NMLE can ensure better quality of admission of physicians and promote reforms in Chinese medical education. There are some experiences of continuous improvement and development of Chinese NMLE, which may have implications for the international community. The first is that Chinese NMLE has effective organizational mechanisms. More specifically, the leadership and organizational structure of NMLE are relatively strong and complete. The director of NHC is the committee director of NMLE. Besides, the directors of provincial and civic health commissions act as group leaders and chief examiners of NMLE. There are some relatively impeccable laws and regulations, such as Law of the People’s Republic of China on Medical Practitioner, Interim Measures of NMLE, Implementation Plan of NMLE, Measures of Handling the Irregularities in NMLE, and Contingency Plan of NMLE, which have been systematically implemented to guarantee the effectiveness of NMLE. In terms of organizational structure, examination districts and testing centers take the grading and administrative responsibility for the organization and implementation of the examination. Moreover, a series of working teams including item development team, examiner team, test management team, information technology team, and research team have been established, providing a strong workforce for NMLE.

In addition, evidence-based reforms and innovations have constantly been made in NMLE. NMEC has gathered a national medical education assessment expert team and funded research projects continuously to improve NMLE. Research on physician’s competence, phased examination, and examination syllabus design based on their competence, test prediction and equation, test validity and reliability, as well as examination fairness have all been conducted [10, 11]. According to the national admission guidelines on physicians, the examination syllabus was defined and the qualification criteria were confirmed. Besides, it also perfected and fine-tuned the best format of medical examinations, embodied humane guidance, and highlighted the orientation of competence [12]. Furthermore, information technology has been applied in NMLE in the past few years. The NMLE has been conducted via computerized modes and a sound information sharing platform has been established. Besides that, the information system of examination management, item bank system, computer test system, and computer-based case simulations system of NMLE have been developed and successfully implemented.

## Main challenges and prospects of NMLE in China

Although these improvements and developments have been achieved, some challenges faced by China’s current NMLE system should also be addressed. Compared with the medical licensing examination of other countries, the component covering the medical humanities (including medical psychology, ethics, and regulation) in NMLE is lower. Similarly, preventive medicine only accounts for 8–10% of the examination syllabus. Furthermore, it might be slightly difficult to assess the competency of examinees’ professionalism, communication skills, and patient care comprehensively by this examination. In addition, the assessment of clinical reasoning ability is not sufficient. Furthermore, the proportion of integrated questions covering both of basic sciences and clinical knowledge is not enough in NMLE.

In accordance with the requirements of the new era and prompt examination results, medical education should improve further and it should pay more attention to the following points.

1. Medical schools should have well-trained teachers, authoritative and innovative teaching materials, high-quality teaching in hospitals, support with sufficient finances and capable managers who have relevant skill sets to further medical education. Medical students should have a heart of benevolence, take interest in medicine, and spend their entire career in medicine.
2. There is an urgent need to encourage medical students to develop morality, high standards, and professionalism in medical education. During the whole process of medical education, priority needs to be accorded to humane medical education and to training physicians with enough knowledge and medical humanism, competence, and professionalism of having reverence for life, healing the wounded and rescuing the dying, and being willing to dedicate their lives for others with social and human welfare zeal. While laying a foundation, medical education for undergraduate students needs to pay attention to the integrity and systematisms of the professional knowledge of medicine. It is necessary to have an organic conformity with regard to various subjects including medical humanism, basic sciences, preventive medicine, clinical medicine, and rehabilitation medicine. Medical education in China needs to embody the principle that the basic sciences are the foundation of clinical medicine and realize the integration of basic and clinical knowledge. In addition, it needs to give expression to overall thinking, broaden the concepts of general medical practice, reflect prevention first, complete the dynamic integration of prevention and cure, demonstrate that humanity is the foundation, and complete an organic integration of humanity and medicine. Medical education should ensure professional competence as the orientation, have educational objectives, and develop a problem-solving approach among students.
3. Medical education further needs to enhance practical teaching and improve the clinical practice competence. Orderly arrangement and gradual deepening of knowledge should be followed during experiment teaching, simulation teaching, and clinical teaching. Medical education needs to include early and repeated clinic multiple times and build systematic clinical thinking. The basic clinical operation skills should be mastered and the concept of medical humanism should also be integrated into medical education and practice.
4. It needs to enhance awareness about public health security. Public health can be considered as population medicine. Public health security involves not only human health but also the national safety and social stability. Prevention is the most economic and effective healthcare strategy, and public health security needs common prevention. It would be a prudent decision to form the idea of prevention first, establish the concept of comprehensive healthcare and integrate prevention, treatment, and health protection.
5. It needs to absorb the essence of human medicine. Traditional medicine is also an important component of medical science. Both Eastern and Western countries have inherited and developed it. The education system thus needs to consider health as the core concern, and have a dynamic integration of modern medicine and traditional medicine to learn from the strong points of both and offset the weakness, thus bringing advantage to the community and entire public and together coping with diseases and injuries.
6. It needs to reinforce international cooperation. Diseases are a common enemy of all human beings and health is a common pursuit of entire humankind. The physicians act as the guardians of human health. Medical education worldwide should have a common dream and pursuit, build medical education standards with common medical values and different national characteristics, promote the development and prosperity of global medical education, make better contributions to the health of humans, and raise the professional standards in China.

To better facilitate medical education development in China, some reforms will be undertaken in NMLE in the future. For the clinical skill examination, more stations should be added to better simulate real clinical settings. The content should cover more medical humanities to comprehensively assess the competency of professionalism, communication skills, and patient care. In addition, computer-based case simulation (CCS) should be considered to assess the examinee’s clinical reasoning ability. The content should cover more disease prevention strategies in the comprehensive medical knowledge examination to better deal with the current public COVID-19 crisis. It should also focus on assessing whether the examinees can apply important concepts of basic sciences to clinical medical practice. Therefore, more case-based questions should be preferred in NMLE. Furthermore, an information management system should be adopted to better implement NMLE nationwide.

## Conclusions

Overall, it is concluded that in China, NMLE needs further retuning. To have a robust medical education system in China and to foster healthy patient-doctor relations, a multipronged, comprehensive strategy is suggested for a better China. This commentary only briefly introduced NMLE to international readers, and more details will be discussed in further research.

## Data Availability

Not applicable.

## Abbreviations

WFME: World Federation for Medical Education
NMLE: National Medical Licensing Examination
NMEC: National Medical Examination Center
NHC: National Health Commission
MCQs: Multiple-choice questions
